# Latent *Toxoplasma gondii* Infection and Associated Risk Factors among HIV-Infected Individuals at Arba Minch Hospital, South Ethiopia

**DOI:** 10.1155/2014/652941

**Published:** 2014-11-09

**Authors:** Tsegaye Yohanes, Serkadis Debalke, Endalew Zemene

**Affiliations:** ^1^Department of Medical Laboratory Science, Arba Minch University, P.O. Box 21, Arba Minch, Ethiopia; ^2^Department of Medical Laboratory Sciences and Pathology, College of Public Health and Medical Sciences, Jimma University, P.O. Box 378, Jimma, Ethiopia

## Abstract

Toxoplasmosis is a parasitic disease caused by *Toxoplasma gondii* (*T. gondii*). The parasite has cosmopolitan distribution, infecting almost all species of warm-blooded animals. Latent *T. gondii* infection in HIV/AIDS patients is a risk for development of cerebral toxoplasmosis (CT). The aim of this study is to determine seroprevalence of latent *T. gondii* infection and assess its associated factors among individuals infected with HIV in Arba Minch Hospital, south Ethiopia. A facility-based cross-sectional study involving 170 HIV-infected individuals attending Arba Minch Hospital antiretroviral therapy (ART) clinic was conducted from April to June 2013. Data on demographic profile of the study participants and factors associated with *T. gondii* infection were gathered using a questionnaire. Serum was tested for IgG anti-*T. gondii* antibody by enzyme-linked immunosorbent assay (ELISA). Data were analyzed using SPSS version 20 software. Seroprevalence of latent *T. gondii* infection among the study participants was 88.2%. Consumption of raw meat (AOR = 4.361; 95% CI: 1.409–13.496) and involvement in farming/gardening activities (AOR = 4.051; 95% CI: 1.112–14.758) were independent predictors of *T. gondii* seropositivity. This study revealed high prevalence of latent *T. gondii* infection, similar to other studies. Monitoring of the patients to prevent reactivation of the latent *T. gondii* infection is recommended.

## 1. Introduction

Toxoplasmosis is a disease caused by* Toxoplasma gondii* (*T. gondii*), an obligate intracellular parasite infecting virtually all species of warm-blooded animals. The parasite has a worldwide distribution with an estimated one-third of the world's population being infected. It has a complex life cycle, undergoing sexual phase in the feline definitive host and asexual phase in its intermediate hosts. The parasite is transmitted to humans mainly by accidental ingestion of its oocysts from cat faeces, consumption of infected raw meat, and rarely vertical transmission during pregnancy [1]. Clinical presentations of the disease are diverse and mainly depend on the immune status of the host. The disease is usually self-limited in immunocompetent individuals, rarely causing pulmonary toxoplasmosis [2]. However, it may result in life-threatening disease, cerebral toxoplasmosis (CT), in immunocompromised individuals [3].

*T. gondii* is one of the opportunistic parasites causing morbidity and mortality in HIV-infected individuals. Primary infection with the parasite initially results in IgM immune response, followed by specific IgG anti-*T. gondii* response. Following immune response of the host against* T. gondii*, cyst stage of the parasite which contains slow replicating bradyzoites forms in skeletal muscles and neuronal tissues. However, in immunocompromised individuals, reactivation of the latent* T. gondii* as a result of conversion of the bradyzoites to the proliferative tachyzoite stage may result in toxoplasmic encephalitis (TE) [4]. The risk of TE increases with decease in CD4+ T lymphocyte count [5].

Several studies documented varying magnitude of latent* T. gondii* infection in HIV-infected individuals. Seroprevalence of* T. gondii* in HIV-infected individuals is often high in most of the reports, with substantial incidence of TE in AIDS patients not receiving prophylaxis [6]. Most of the available studies on magnitude of* T. gondii* infection in HIV-infected individuals in Ethiopia reported prevalence of more than 75% [7–10]. Moreover,* T. gondii* seroprevalence of 81.4% among women of child bearing age in central Ethiopia [11] and 83.6% in pregnant women in southwest Ethiopia [12] were also documented. Seroprevalence of* T. gondii* is similarly high in most of the sub-Saharan African countries [13–15].

Laboratory diagnosis of* T. gondii* infection in HIV-infected individuals is not a routine practice in health care facilities in Ethiopia. Moreover, data on seroprevalence of* T. gondii* is limited in Ethiopia, and published reports on magnitude of* T. gondii* infection in HIV-infected individuals in the study area have not been obtained. Therefore, this study determined seroprevalence of latent* T. gondii* infection and assessed associated risk factors among HIV-infected individuals attending Arba Minch Hospital antiretroviral therapy (ART) clinic.

## 2. Methods

### 2.1. Study Design and Setting

A facility-based cross-sectional study was conducted in Arba Minch Hospital found in Arba Minch town. The town is located 505 km south of the capital Addis Ababa. Arba Minch is located at altitude of 1200–1300 meters above sea level, with average annual temperature of 29°C. The hospital serves more than 2 million people in the region. The study was conducted from April to June 2013.

### 2.2. Study Population and Sampling

A total of 170 HIV seropositive individuals who were on follow-up at Arba Minch Hospital ART clinic during the months April to June 2013 were included in the study. The study participants were prior diagnosed with HIV and under follow-up at the ART clinic of the hospital. The sample size was estimated using the general formula for single population proportion, with the following assumptions: prevalence (p) of 87.4% [8], margin of error of 0.05, and confidence level of 95%. Accordingly, a total of 170 HIV seropositive individuals visiting Arba Minch Hospital ART clinic were included in the study. Study participants visiting the ART clinic during the study period were enrolled consecutively. At the beginning of data collection, a total of 1,650 HIV-infected individuals were registered at the ART clinic of the hospital.

### 2.3. Data Collection

A pretested questionnaire was used to gather sociodemographic information and data on factors predisposing to* T. gondii* infection. The questions administered for assessing predisposing factors of* T. gondii *infection include habit of eating raw meat and vegetables/fruits, farming/gardening activities, sources of drinking water, history of blood transfusion, and presence of domestic cats at household. The questionnaire data were collected by a trained nurse. Following the interview, approximately 2 mL of venous blood was collected from each consenting study participant by experienced phlebotomist. The blood was processed following standard procedures. Briefly, serum was separated from red blood cells and stored at −20°C prior to assay. It was then tested for anti-*T. gondii *IgG antibody using ELISA test kit (*Human Gesellschaft für Biochemica und Diagnostica mbH*, Wiesbaden, Germany) at Arba Minch Regional Laboratory Department, following the manufacturer's instruction. The CD4+ T cell count of the study participants was obtained from log book of the ART clinic.

### 2.4. Data Analyses

Data were first collected in hardcopy. After checking for completeness and consistency, the data were entered into computer, cleaned, and analyzed using SPSS version 20.0 software package. Descriptive statistics were performed to describe demographic profile of the study participants. Bivariate and multivariate logistic regression analyses were utilized to identify factors predisposing to* T. gondii* infection. Variables with *P* value <0.25 by the bivariate analysis were entered into multivariate model. A *P* value <0.05 was set as statistically significant during the analysis.

### 2.5. Ethical Considerations

Ethical clearance was obtained from Jimma University Research Ethical Review Committee. Official permission was sought from Arba Minch Zonal Health Bureau and Arba Minch Hospital Administration. Moreover, written informed consent was obtained from all study participants prior to enrollment in the study. Confidentiality of the collected information and laboratory test results was maintained. Laboratory test results were communicated to the attending physician for further management of the cases.

## 3. Results

### 3.1. Sociodemographic Characteristics of the Study Participants

A total of 170 HIV seropositive individuals attending Arba Minch Hospital ART clinic had participated in this study. The majority (64.1%) of the study participants were female. Mean age of the participants was 35.54 years. The vast majority of the study participants (86.5%) were urban residents. Sociodemographic profile of the study participants is demonstrated in Table 1.

### 3.2. Seroprevalence of* T. gondii* Infection and Associated Factors

Overall, out of the total 170 HIV seropositive study participants included in this study, 150 (88.2%) were IgG anti-*T. gondii *antibody seropositive.

Several sociodemographic and other factors predisposing to* T. gondii* infection were also assessed. More than one-third (37.6%) of the study participants were within the age group 35–44 with* T. gondii* seroprevalence of 90.6%. IgG anti-*T. gondii* seropositivity increased as age group of the study participants increases. The difference was significant (Table 2). In this study, 64.1% of the study participants were female.* T. gondii* seroprevalence among the female participants was 89%.* T. gondii* infection was not significantly associated (*P* > 0.05) with gender of the study participants. Similarly, no significant difference in latent* T. gondii* infection with place of residence, educational status, consumption of raw vegetables or fruits, source of drinking water, and history of blood transfusion was observed in this study (Table 2).

Data on CD4+ T lymphocyte count were available for 136 of the study participants. The CD4+ T cell count ranged from 55 to 1574 cells/*μ*L. IgG anti-*T. gondii* seropositivity among study participants with CD4+ T cell count below 200 and ≥200 cells/*μ*L was 84.6% and 89.4%, respectively. The difference in latent* T. gondii* infection with CD4+ T cell count was not significant (*P* < 0.05) (Table 2).

Multivariate logistic regression model was used to identify predictors of latent* T. gondii* infection (Table 2). Accordingly, habit of eating raw meat (AOR = 4.4, 95% CI: 1.409–13.496) and involvement in farming/gardening activities (AOR = 4.1, 95% CI: 1.112–14.758) were predictors for latent* T. gondii* infection in this study.

## 4. Discussion

In this study, seroprevalence of anti-*Toxoplasma gondii *IgG antibody among the HIV positive study participants was 88.2%.* T. gondii* is an important opportunistic parasitic infection in HIV-infected individuals. Primary infection with* T. gondii *results in initial IgM anti-*T. gondii* antibody response, followed by IgG antibody, which apparently remains for life. Detection of specific IgG anti-*T. gondii* antibody, therefore, indicates chronic infection with the parasite. Chronic* T. gondii* infection in HIV-infected individuals is a risk for development of CT [16], especially when CD4+ T lymphocyte count falls below 100 cells/*μ*L [17]. The high prevalence of chronic* T. gondii* infection in our study participants, therefore, highlights the need for prevention of CT [18]. This requires regular monitoring of the HIV-infected individuals and treatment of the eligible ones [3]. It is likely that* T. gondii* is also highly prevalent in unrecognized HIV-infected individuals, putting them at high risk of developing CT. An earlier report documented low utilization of HIV counseling and testing among men in Ethiopia [19]. Cerebral toxoplasmosis is one of the central nervous system disorders and AIDS-defining opportunistic infections in HIV/AIDS patients [20–23].

Screening of HIV-infected individuals for* T. gondii* infection is not a routine practice in health care centers in Ethiopia. The high prevalence of chronic* T. gondii* infection in the study participants alerts health care professionals to consider this opportunistic parasite to prevent the neurological complications associated with it. The high prevalence of latent* T. gondii* infection in the HIV-infected individuals in our study is consistent with previous reports from Bahir Dar [8] and Addis Ababa [7], in which seroprevalence of 87.4% and 93.3% was documented, respectively. In contrast, relatively low prevalence of* T. gondii* in HIV-infected individuals was documented elsewhere [24, 25].

Consumption of raw meat is one of the risk factors of* T. gondii* infection. In this study, raw meat consumption was significantly associated with* T. gondii* infection and is one of the predictors of the infection. Study participants who consume raw meat were four times more likely to be* T. gondii* seropositive. Some studies also reported significant association of consumption of raw meat with* T. gondii* infection in human population [26–29], whereas others [9, 12, 30] documented no significant association. The rate of* T. gondii* infection in the animal population may account for the difference. For example, only less than 3% of the cattle in a study done in Brazil [31] were* T. gondii* seropositive as compared to seroprevalence of 22.9% in the sheep, 11.6% in goats, and 6.6% in cattle in an earlier study from central Ethiopia [32]. A recent review [33] reported that 79% of the sheep and goats in Ethiopia had serological evidence of* T. gondii* infection.

The other predictor of* T. gondii* seropositivity in this study is involvement in farming/gardening activities. In Ethiopia, it is very likely that soil can easily be contaminated by cat faeces, as it is common to find stray cats everywhere around human habitation. As infected cats excrete millions of environmentally resistant oocysts of the parasite within short period of time, they play an important role in disseminating the parasite. Recently, oocysts were isolated from nearly one in five of feral cats in Addis Ababa area [34]. The oocysts can survive for months in the soil especially under damp conditions [35]. The significantly higher proportion of the study participants involved in farming/gardening activities being* T. gondii* seropositive is likely related to improper washing of hands after the activities and before meal.

Despite the significant association of farming/gardening activities with* T. gondii* seropositivity, it appears that presence of domestic cat(s) in the household did not significantly affect* T. gondii* seropositivity. Presence of domestic cats at household as a risk factor was documented in school children elsewhere [36]. Difference in infection rate of the cats in these areas may account for the difference.

Blood transfusion is also a possible means of transmission of* T. gondii*. In the current study, 6.5% of the study participants responded to having had previous blood transfusion, 90.9% of whom were* T. gondii* seropositive. There was no significant difference in* T. gondii* seropositivity with history of blood transfusion. The risk of* T. gondii* infection through blood transfusion is low, probably due to the low prevalence of acute* T. gondii* infection in blood donors.

In this study, CD4 + T lymphocyte count of 80% of the study participants was available during the data collection period. There was no significant difference in* T. gondii* seropositivity between study participants with CD4 T-cell count below 200 and ≥200 cells/*μ*L. This finding is consistent with a report from Malaysia [37]. In the current study, nearly 10% of the study participants had CD4+ T lymphocyte count less than 200 cells/*μ*L. The vast majority of these were IgG anti-*T. gondii* seropositive. HIV-infected individuals with low CD4+ T cell count coinfected with* T. gondii* are at higher risk of reactivating the latent infection [3]. It has been documented that most cases of TE in AIDS patients are due to reactivation of latent* T. gondii* infection, and incidence of the disease is associated with* T. gondii *IgG seropositivity and low CD4+ T cell count [5].

Among the sociodemographic and other factors assessed in this study, only age was significantly associated with* T. gondii* seropositivity. As age group of the study participants increases, a corresponding increase in* T. gondii* seropositivity was noted. This is probably related to prolonged exposure time as age increases.

## 5. Conclusions

In conclusion, seroprevalence of latent* T. gondii* infection is high among the study participants, similar to most of the studies. The rate of* T. gondii* seropositivity significantly increased with age. Self-reported consumption of raw meat and history of involvement in farming/gardening activities were the main predictors of* T. gondii* seropositivity among the study participants. Creating awareness about* T. gondii *infection and follow-up of their status is recommended. Moreover, screening of* T. gondii* infection in HIV-infected individuals should be considered. Further studies are required to determine incidence of TE in HIV-infected individuals in the area.

## Figures and Tables

**Table 1:** Demographic profile and seroprevalence of latent *Toxoplasma gondii* infection among the study participants (*n* = 170) in Arba Minch Hospital, 2013.

Demographic variables	Seroprevalence		Total *n* (%)
	Positive *n* (%)	Negative *n* (%)	
Age group (years)			
≤24	11 (61.1)	7 (38.9)	18 (10.6)
25–34	48 (90.6)	5 (9.4)	53 (31.2)
35–44	58 (90.6)	6 (9.4)	64 (37.6)
≥45	33 (94.3)	2 (5.7)	35 (20.6)
Sex			
Male	53 (86.9)	8 (13.1)	61 (35.9)
Female	97 (89.0)	12 (11.0)	109 (64.1)
Place of residence			
Urban	129 (87.8)	18 (12.2)	147 (86.5)
Rural	21 (91.3)	2 (8.7)	23 (13.5)
Marital status			
Single	30 (88.2)	4 (11.8)	34 (20.0)
Married	79 (87.8)	11 (12.2)	90 (52.9)
Divorced	14 (82.4)	3 (17.6)	17 (10.0)
Widowed	27 (93.1)	2 (6.9)	29 (17.1)
Educational status			
Illiterate	36 (83.7)	7 (16.3)	43 (25.3)
Primary	69 (92.0)	6 (8.0)	75 (44.1)
Secondary	34 (85.0)	6 (15.0)	40 (23.5)
Tertiary	11 (91.7)	1 (8.3)	12 (7.1)
Occupational status			
Employed	81 (89.0)	10 (11.0)	91 (53.5)
Housewives	46 (90.2)	5 (9.8)	51 (30.0)
Others^*^	23 (82.1)	5 (17.9)	28 (16.5)

^*^Students, farmers, house maids, and daily laborers.

**Table 2:** Factors associated with *T. gondii* infection in the study participants, Arba Minch Hospital, 2013.

Characteristics	Seroprevalence		COR (95% CI)	AOR (95% CI)
	Positive *n* (%)	Negative *n* (%)		
Age group (years)				
≤24	11 (61.1)	7 (38.9)	1	1
25–34	48 (90.6)	5 (9.4)	6.109 (1.630–22.903)	6.266 (1.479–26.539)^*^
35–44	58 (90.6)	6 (9.4)	6.152 (1.733–21.832)	7.176 (1.675–30.748)^*^
≥45	33 (94.3)	2 (5.7)	10.500 (1.893–58.242)	7.205 (1.040–49.932)^*^
Sex				
Male	53 (86.9)	8 (13.1)	0.820 (0.315–2.130)	
Female	97 (89.0)	12 (11.0)	1	
Place of residence				
Urban	129 (87.8)	18 (12.2)	1	
Rural	21 (91.3)	2 (8.7)	1.465 (0.317–6.779)	
Educational status				
Illiterate	36 (83.7)	7 (16.3)	0.586 (0.217–1.582)	
Literate	114 (89.8)	13 (10.2)	1	
Habit of eating raw meat				
No	25 (69.4)	11 (30.6)	1	1
Yes	125 (93.3)	9 (6.7)	6.111 (2.294–16.283)	4.361 (1.409–13.496)^*^
Eating raw vegetable or fruits				
No	70 (87.5)	10 (12.5)	1	
Yes	80 (88.9)	10 (11.1)	1.143 (0.449–2.906)	
Presence of cat(s) at home				
No	107 (86.3)	17 (13.7)	1	1
Yes	43 (93.5)	3 (6.5)	2.277 (0.635–8.169)	3.417 (0.743–15.717)
Farming/gardening activity				
No	78 (83.9)	15 (16.1)	1	1
Yes	72 (93.5)	5 (6.5)	2.769 (0.958–8.006)	4.051 (1.112–14.758)^*^
Source of drinking water				
Well	13 (76.5)	4 (23.5)	0.380 (0.110–1.304)	0.237 (0.048–1.165)
Pipe	137 (89.5)	16 (10.5)	1	1
History of blood transfusion				
No	140 (88.1)	19 (11.9)	1	
Yes	10 (90.9)	1 (9.1)	1.357 (0.164–11.202)	
CD4+ T-lymphocyte count per *μ*L (*n* = 136)				
<200	11 (84.6)	2 (15.4)	0.650 (0.130–3.260)	
≥200	110 (89.4)	13 (10.6)	1	

^*^Variables significant by the multivariate analysis.

AOR: adjusted odds ratio, adjusted for other variables in the table; CI: confidence interval; COR: crude odds ratio.

## References

[1] Dubey J. P. (2010). *Toxoplasmosis of Animals and Humans*.

[2] Leal F. E., Cavazzana C. L., de Andrade H. F., Galisteo A. J., de Mendonça J. S., Kallas E. G. (2007). *Toxoplasma gondii* pneumonia in immunocompetent subjects: case report and review. *Clinical Infectious Diseases*.

[3] Kodym P., Malý M., Beran O., Jilich D., Rozsypal H., Machala L., Holub M. (2014). Incidence, immunological and clinical characteristics of reactivation of latent *Toxoplasma gondii* infection in HIV-infected patients. *Epidemiology and Infection*.

[4] Luma H. N., Tchaleu B. C. N., Mapoure Y. N., Temfack E., Doualla M. S., Halle M. P., Joko H. A., Koulla-Shiro S. (2013). Toxoplasma encephalitis in HIV/AIDS patients admitted to the Douala general hospital between 2004 and 2009: a cross sectional study. *BMC Research Notes*.

[5] Djurković-Djaković O., Bobić B., Vuković D., Marinković J., Jevtović D. (1997). Risk for toxoplasmic encephalitis in AIDS patients in Yugoslavia. *International Journal of Infectious Diseases*.

[6] Nascimento L. V., Stollar F., Tavares L. B., Cavasini C. E., Maia I. L., Cordeiro J. A., Ferreira M. U. (2001). Risk factors for toxoplasmic encephalitis in HIV-infected patients: a case-control study in Brazil. *Annals of Tropical Medicine and Parasitology*.

[7] Shimelis T., Tebeje M., Tadesse E., Tegbaru B., Terefe A. (2009). Sero-prevalence of latent *Toxoplasma gondii* infection among HIV-infected and HIV-uninfected people in Addis Ababa, Ethiopia: a comparative cross-sectional study. *BMC Research Notes*.

[8] Walle F., Kebede N., Tsegaye A., Kassa T. (2013). Seroprevalence and risk factors for Toxoplasmosis in HIV infected and non-infected individuals in Bahir Dar, Northwest Ethiopia. *Parasites & Vectors*.

[9] Muluye D., Wondimeneh Y., Belyhun Y. (2013). Prevalence of *Toxoplasma gondii* and associated risk factors among people living with HIV at Gondar University Hospital, Northwest Ethiopia. *ISRN Tropical Medicine*.

[10] Aleme H., Tilahun G., Fekade D., Berhe N., Medhin G. (2013). Sereoprevalence of immunoglobulin-G and of immunoglobulin-M anti- Toxoplasma gondii a ntibodies in human immunodeficiency virus infection/acquired immunodeficiency syndrome patients at Tikur Anbessa Specialized Hospital, Addis Ababa, Ethiopia. *Journal of Infectious Diseases and Therapy*.

[11] Gebremedhin E. Z., Abebe A. H., Tessema T. S., Tullu K. D., Medhin G., Vitale M., Di Marco V., Cox E., Dorny P. (2013). Seroepidemiology of Toxoplasma gondii infection in women of child-bearing age in central Ethiopia. *BMC Infectious Diseases*.

[12] Zemene E., Yewhalaw D., Abera S., Belay T., Samuel A., Zeynudin A. (2012). Seroprevalence of *Toxoplasma gondii* and associated risk factors among pregnant women in Jimma town, Southwestern Ethiopia. *BMC Infectious Diseases*.

[13] Ayi I., Edu S. A., Apea-Kubi K. A. (2009). Sero-epidemiology of toxoplasmosis amongst pregnant women in the greater Accra region of Ghana. *Ghana Medical Journal*.

[14] Addebbous A., Adarmouch L., Tali A., Laboudi M., Amine M., Aajly L., Rhajaoui M., Chabaa L., Zougaghi L. (2012). IgG anti-toxoplasma antibodies among asymptomatic HIV-infected patients in Marrakesh-Morocco. *Acta Tropica*.

[15] Lindström I., Kaddu-Mulindwa D. H., Kironde F., Lindh J. (2006). Prevalence of latent and reactivated *Toxoplasma gondii* parasites in HIV-patients from Uganda. *Acta Tropica*.

[16] Zangerle R., Allerberger F., Pohl P., Fritsch P., Dierich M. P. (1991). High risk of developing toxoplasmic encephalitis in AIDS patients seropositive to *Toxoplasma gondii*. *Medical Microbiology and Immunology*.

[17] George S. M., Malik A. K., Al Hilli F. (2009). Cerebral toxoplasmosis in an HIV positive patient: a case report and review of pathogenesis and laboratory diagnosis. *Bahrain Medical Bulletin*.

[18] Centers for Disease Control and Prevention (2009). *Guidelines for Prevention and Treatment of Opportunistic Infections in HIV-Infected Adults and Adolescents*.

[19] Leta T. H., Sandøy I. F., Fylkesnes K. (2012). Factors affecting voluntary HIV counselling and testing among men in Ethiopia: a cross-sectional survey. *BMC Public Health*.

[20] Dai L., Mahajan S. D., Guo C., Zhang T., Wang W., Li T., Jiang T., Wu H., Li N. (2014). Spectrum of central nervous system disorders in hospitalized HIV/AIDS patients (2009–2011) at a major HIV/AIDS referral center in Beijing, China. *Journal of the Neurological Sciences*.

[21] Balkhair A. A., Al-Muharrmi Z. K., Ganguly S., Al-Jabri A. A. (2012). Spectrum of AIDS defining opportunistic infections in a series of 77 hospitalised HIV-infected Omani patients. *Sultan Qaboos University Medical Journal*.

[22] Berhe T., Melkamu Y., Amare A. (2012). The pattern and predictors of mortality of HIV/AIDS patients with neurologic manifestation in Ethiopia: a retrospective study. *AIDS Research and Therapy*.

[23] Amogne W., Teshager G., Zenebe G. (2006). Central nervous system toxoplasmosis in adult ethiopians. *Ethiopian Medical Journal*.

[24] Ramírez M. D. L. L. G., Alvarado V. V., Gutierrez G. V., González O. J., Cosio C. G., Sandoval M. V. (1997). Prevalence of IgG and IgM anti-*Toxoplasma* antibodies in patients with HIV and acquired immunodeficiency syndrome (AIDS). *Revista da Sociedade Brasileira de Medicina Tropical*.

[25] Nissapatorn V., Lee C., Quek K. F., Leong C. L., Mahmud R., Abdullah K. A. (2004). Toxoplasmosis in HIV/AIDS patients: a current situation. *Japanese Journal of Infectious Diseases*.

[26] Alvarado-Esquivel C., Pacheco-Vega S. J., Hernández-Tinoco J. (2014). Seroprevalence of *Toxoplasma gondii* infection and associated risk factors in Huicholes in Mexico. *Parasites & Vectors*.

[27] Jones J. L., Dargelas V., Roberts J., Press C., Remington J. S., Montoya J. G. (2009). Risk factors for *Toxoplasma gondii* infection in the United States. *Clinical Infectious Diseases*.

[28] Kolbekova P., Kourbatova E., Novotna M., Kodym P., Flegr J. (2007). New and old risk-factors for *Toxoplasma gondii* infection: prospective cross-sectional study among military personnel in the Czech Republic. *Clinical Microbiology and Infection*.

[29] Studeničová C., Benčaiová G., Holková R. (2006). Seroprevalence of *Toxoplasma gondii* antibodies in a healthy population from Slovakia. *European Journal of Internal Medicine*.

[30] Minbaeva G., Schweiger A., Bodosheva A., Kuttubaev O., Hehl A. B., Tanner I., Ziadinov I., Torgerson P. R., Deplazes P. (2013). *Toxoplasma gondii* infection in Kyrgyzstan: seroprevalence , risk factor analysis, and estimate of congenital and AIDS-related toxoplasmosis. *PLoS Neglected Tropical Diseases*.

[31] Fajardo H. V., D'Ávila S., Bastos R. R., Cyrino C. D., De Lima Detoni M., Garcia J. L., Das Neves L. B., Nicolau J. L., Amendoeira M. R. R. (2013). Seroprevalence and risk factors of toxoplasmosis in cattle from extensive and semi-intensive rearing systems at Zona da Mata, Minas Gerais state, Southern Brazil. *Parasites and Vectors*.

[32] Bekele T., Kasali O. B. (1989). Toxoplasmosis in sheep, goats and cattle in central Ethiopia. *Veterinary Research Communications*.

[33] Dubey J. P., Tiao N., Gebreyes W. A., Jones J. L. (2012). A review of toxoplasmosis in humans and animals in Ethiopia. *Epidemiology and Infection*.

[34] Dubey J. P., Darrington C., Tiao N. (2013). Isolation of viable *Toxoplasma gondii* from tissues and feces of cats from Addis Ababa, Ethiopia. *Journal of Parasitology*.

[35] Lélu M., Villena I., Dardé M.-L., Aubert D., Geers R., Dupuis E., Marnef F., Poulle M.-L., Gotteland C., Dumètre A., Gilot-Fromonta E. (2012). Quantitative estimation of the viability of *Toxoplasma gondii* oocysts in soil. *Applied and Environmental Microbiology*.

[36] Lopes F. M. R., Gonçalves D. D., Dos Reis C. R., Breganó R. M., Freire R. L., de Freitas J. C., Navarro I. T. (2008). Presence of domesticated cats and visual impairment associated to Toxoplasma gondii serum positive children at an elementary school in Jataizinho, state of Paraná, Brazil. *Revista Brasileira Parasitologia Veterinária*.

[37] Nissapatorn V., Kamarulzaman A., Init I., Tan L. H., Rohela M., Norliza A., Chan L. L., Latt H. M., Khairul Anuar A., Quek K. F. (2002). Seroepidemiology of toxoplasmosis among HIV-infected patients and healthy blood donors. *Medical Journal of Malaysia*.

